# Cohort profile: the *Kisalaya* cohort of mother-infant dyads in rural south India (2008-2012)

**DOI:** 10.4178/epih.e2020010

**Published:** 2020-03-11

**Authors:** Smitha Chandrashekarappa, Krupa Modi, Karl Krupp, Kavitha Ravi, Anisa Khan, Vijaya Srinivas, Poornima Jaykrishna, Anjali Arun, Murali Krishna, Purnima Madhivanan

**Affiliations:** 1 Department of Community Medicine, JSS Medical College, JSS Academy of Higher Education and Research, Mysuru, India; 2 Public Health Research Institute of India (PHRII), Mysuru, India; 3 University of California, Berkeley, CA, USA; 4 Department of Health Promotion Sciences, University of Arizona, Mel & Enid Zuckerman College of Public Health, Tucson, AZ, USA; 5 Foundation for Research and Advocacy in Mental Health (FRAMe), Mysuru, India; 6 Department of Medicine & Department of Family Community Medicine, University of Arizona College of Medicine, Tucson, AZ, USA

**Keywords:** Pregnant women, Antenatal care, Cohort profile, Longitudinal birth cohort, Rural, India

## Abstract

The *Kisalaya* cohort was established in 2008, providing integrated antenatal care (ANC) and human immunodeficiency virus (HIV) testing in order to reduce adverse birth outcomes and pediatric HIV infections. The program used a mobile clinic model to deliver health education, ANC, and HIV/sexually transmitted infection testing and management to pregnant women in rural communities in southern India. This cohort includes pregnant women residing in 144 villages of the Mysuru *taluk* (a rural region) who received ANC through the mobile clinic and delivered their infants between 2008 and 2011. Of the 1,940 women registered for ANC at primary healthcare centers during this time period, 1,675 (75.6%) were enrolled in the *Kisalaya* cohort. Once women enrolled in the *Kisalaya* cohort gave birth, the cohort expanded to include the mother-infant dyads with a retention rate of 100% at follow-up visits at 15 days and at 6 months post-delivery. The baseline data collected during the *Kisalaya* study included both questionnaire-based data and laboratory-based investigations. Presently, a study entitled “*Early life influences on adolescent mental health: a life course study of the Kisalaya birth cohort in south India*” is in the process of data collection (2019-2020).

## INTRODUCTION

### Why was it set up?

**Figure 1.**
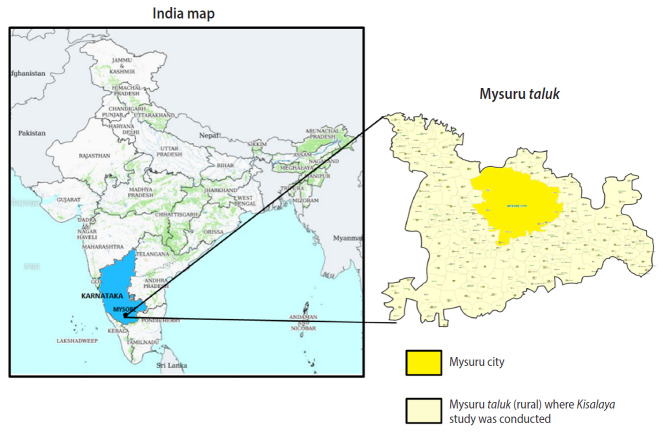
Picture depicting the Mysuru *taluk* rural area where the *Kisalaya* study was conducted. Source from: Government of India. Indian space research organization (ISRO) centres [7].

Since the early 2000s, India has made significant strides in the health sector, as indicated by major reductions in infant mortality and maternal mortality rates [1-4]. Nonetheless, the country struggles with addressing chronic diseases, nutritional deficiencies, newborn health outcomes, women’s health issues, and sexually transmitted and blood-borne infection rates. A large challenge in addressing these health outcomes is the lack of access to quality healthcare among vulnerable and remote populations, such as rural and tribal communities, especially considering that the vast majority of Indians live in rural areas [5,6]. In an effort to address health disparities in rural areas, the *Kisalaya* (“young tender shoots”) cohort was established in 2008, providing integrated antenatal care (ANC) and human immunodeficiency virus (HIV) testing in order to reduce adverse birth outcomes and pediatric HIV infections. The program used a mobile clinic model to deliver health education, ANC, and HIV/sexually transmitted infection (STI) testing and management to pregnant women in 144 rural villages in the Mysuru *taluk* of Mysuru District (Figure 1) [7]. The mother-offspring dyads were followed-up at 15 days and 6 months post-delivery. The study team continues to stay connected to the dyads informally on a regular basis. This cohort design allows investigators to monitor how access to comprehensive prenatal and HIV services impact postnatal health outcomes for both women and children over time. The rich data pertaining to maternal and child health will be used in our future research related to the developmental origins of health and disease (DOHaD) model of cognition and cardiometabolic risk factors among the offspring.

### Who is in the cohort?

This cohort includes pregnant women residing in 144 villages of the Mysuru *taluk* who received ANC through the mobile clinic and delivered their children between 2008 and 2011. In total, 1,940 women registered for ANC at primary healthcare centers through the Reproductive and Child Health Program of the government of India during this time period and 274 were additionally identified by the research team. Of the women registered with the program, 1,675 (75.6%) agreed to participate in the baseline study that resulted in the *Kisalaya* cohort. No specific sampling method was used for the study, as all pregnant women in the geographical area who resided in the rural Mysuru *taluk* were eligible and invited to participate. Later the registered antenatal women who provided informed consent were included in the study. By integrating ANC and HIV prevention services into a one-stop mobile health clinic model, the program was able to deliver health education, ANC, and HIV prevention services at the doorsteps of the women.

**Figure 2.**
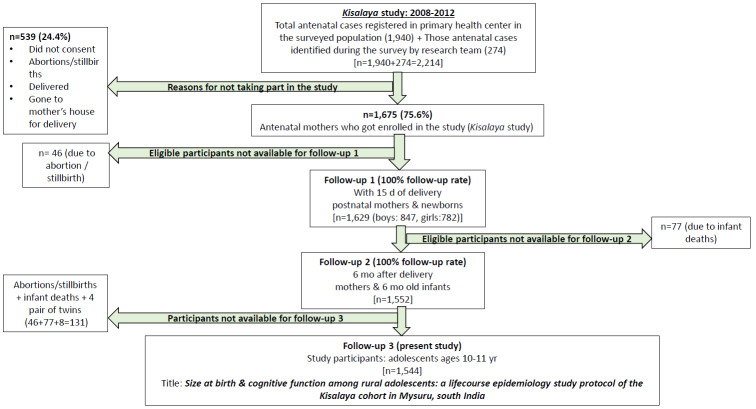
Depicting the follow-up of *Kisalaya* cohort.

In order to support the program, traditional birth attendants (TBAs) were identified by location and trained to accompany the pregnant women to the mobile clinics. Furthermore, to assist with the delivery of services in the community, frontline community health workers (CHW), including accredited social health activists (ASHAs) and auxiliary nurse midwifes (ANMs), in rural regions of the Mysuru *taluk* were trained on topics related to perinatal mortality and morbidity, issues and barriers related to institutional deliveries, counseling and testing for HIV, prevention of vertical transmission of HIV, and management of pregnancy-related complications. Mobile medical clinics were then used to extend health services to pregnant women in hard-to-reach areas. Once the women enrolled in the *Kisalaya* cohort gave birth, the cohort expanded to include the mother-infant dyads, with a retention rate of 100% for follow-up visits at 15 days (n= 1,629) and at 6 months (n= 1,552) post-delivery (accounting for 77 infant deaths that took place between the 2 follow-up points) (Figure 2). The extent of follow-up of the offspring is also an indicator of the success of the mobile maternal services that were provided for these rural women [8].

## STUDY PARTICIPANTS

### How often has the cohort been followed-up?

The *Kisalaya* cohort was established in 2008, when initial ANC visits were conducted with pregnant women. ANC visits were conducted regularly between 2008 and 2011 using mobile medical clinics. All study subjects were later followed-up during the postnatal period, within 15 days of giving birth, and 6 months after birth during 2008-2012. Although no data were collected from the cohort between 2012 to 2019 due to lack of funding, the research team at the Public Health Research Institute of India (PHRII) has stayed in contact with the families of the mother-infant dyads who constituted the cohort, as well the communities in which the study was conducted.

Presently, a study entitled “*Early life influences on adolescent mental health: a life course study of the Kisalaya birth cohort in south India*” is ongoing between 2019 and 2020. The study will retrace the mother-child dyads of the *Kisalaya* cohort to explore the role of early life influences on adolescent mental health. The children, who are now 10-12 years old, will undergo mental health assessments and evaluations of their cognitive ability (data set IV, Table 1). This data will then be linked to previously collected information, including demographic data, details about the mother’s pregnancy, and newborn health outcomes. We have now successfully retraced the cohort and all singleton offspring have been invited to participate in the ongoing DOHaD studies of cognition and brain health. There are no specific exclusion criterion for the offspring and we have not devised any specific sampling strategy, as we would like to examine all the offspring to establish as large a cohort of adolescents as possible.

### Ethics statement

This study was conducted according to the guidelines laid down in the Declaration of Helsinki and all procedures involving human subjects/patients were approved by the Institutional Review Board of Vikram Hospital (2008-04-12-01), Mysuru, India. All women underwent the informed consent process before participating in the study. Additional reviews and approvals were received from the Institutional Review Board at Florida International University, FL, USA.

## MEASUREMENTS

### What has been measured?

The data collected during the 2008-2011 *Kisalaya* program include both observational data obtained through interviewer-administered questionnaires and biomedical data from laboratory investigations using serum, urine, and vaginal swabs. Questionnaires were administered in the local language (Kannada). The mean age of the mothers at baseline was 20.88± 2.82 years, and 98.7% reported their religion as Hindu, while 1.3% were Muslims. Most (97.6%) women said they were housewives and the remaining women were engaged in a skilled occupation. Slightly more than one-eighth of the women (13.4%) were reported as illiterate. The follow-up visits after childbirth showed that 48.0% (n= 782) of women gave birth to girls and 52.0% (n= 847) to boys. There were 46 abortions and stillbirths that could not be followed-up for postnatal outcomes at the first visit at 15 days postpartum. Additionally, 77 children died after the 15-day visit and could not be followed-up at the 6-month visit. Hence, of the total of 1,675 pregnancies enrolled in the *Kisalaya* program, 123 singleton pregnancies (due to abortions, stillbirths, and post-delivery deaths) and 4 pairs of twins will be excluded from the current study, leaving 1,544 mother-child dyads available to be retraced.

#### During the antenatal period

The data collected included demographic information, socioeconomic status, details of the current pregnancy, previous obstetric history, and information about each woman’s main sex partner. Blood, urine, and vaginal swabs were collected using standardized procedures to assess various parameters (data set I, Table 1).

#### Within 15 days postpartum

The data collected included information on breastfeeding, birth preparedness, and details about the delivery and health of the newborn child (data set II, Table 1).

#### At 6 months postpartum

Data were collected about breastfeeding practices, immunization history, and the health of the mother and the child (data set III, Table 1).

## WHAT HAS IT FOUND? KEY FINDINGS AND PUBLICATIONS

The *Kisalaya* cohort was designed to examine the acceptability of integrated HIV/STI testing with routine ANC provided by a mobile medical clinic in a rural setting. It included a community education and awareness component to better engage community members and village stakeholders. The *Kisalaya* longitudinal cohort found this holistic model to be highly successful in promoting comprehensive ANC and prevention of mother-to-child transmission (PMTCT) of HIV services [8].

### Formative work

In order to learn how best to promote uptake of integrated PMTCT services and ANC, a preliminary population-based survey was conducted using a 2-stage probability proportional to size sampling of residents in 16 villages. The residents selected for this study answered an interviewer-administered survey about ANC and institutional delivery practices [9]. The study found that the number of women receiving ANC and having institutional deliveries had greatly increased since the early 2000s, but large disparities among different castes continued to exist. Mothers belonging to general castes were almost twice as likely to have an institutional delivery than women belonging to lower castes such as Scheduled Castes and Scheduled Tribes (SC/ST). Furthermore, women belonging to SC/ST reported lower rates of receiving ANC. Thus, focusing on the provision of ANC and maternal health services in marginalized groups became of critical importance [9].

### Mobile medical clinic outcomes

During the 3-year project, 92 mobile medical clinics were held in 144 villages located outside of the 10 km radius around Mysuru city. Of the 1,940 pregnant women in the 144 rural villages, 1,675 women (76.0%) received ANC from the *Kisalaya* program, with 1,639 (97.8%) of them consenting to HIV testing and counseling, suggesting that women in rural areas will indeed take advantage of reproductive health services when they are made physically available in a culturally sensitive manner.

To explore HIV stigma and fear among the cohort, a nested mixed-methods study investigated the predictive abilities of the social-ecological and maternal-fetal protection models, finding that reproductive life history factors, along with individual, interpersonal, and institutional factors were significant indicators of stigma and fear [10].

At the 15-day and 6-month follow-ups, the *Kisalaya* cohort was able to achieve a 100% follow-up rate, indicating that it is feasible to follow-up mother-infant dyads in a rural setting if robust relationships are developed during the antenatal period.

### Clinical outcomes

All women (100%) who consented to HIV testing received their test results and post-test counseling. Fourteen (0.84%; 95% confidence interval [CI], 0.50 to 1.40) women were seropositive for HIV. These women received management to prevent vertical transmission and were accompanied to a tertiary care facility to receive antiretroviral treatment. Six other women (0.40%; 95% CI, 0.17 to 0.78) were diagnosed with hepatitis B infection, 2 (0.12%; 95% CI, 0.03 to 0.44) were diagnosed with syphilis, and 112 (6.70%; 95% CI, 5.59 to 8.00) had herpes simplex virus type 2 antibodies [11]. The presence of these infections in pregnant women indicated that education and awareness efforts about prevention must involve both men and women. Ninety-seven women (5.90%; 95% CI, 4.70 to 7.00) were also diagnosed with bacterial vaginosis, which is a known risk factor for adverse birth outcomes.

Pregnant women were screened for other non-communicable conditions that are important during pregnancy, and 5.7% (95% CI, 4.7 to 6.9) had gestational hypertension, 18.7% (95% CI, 16.9 to 20.6) were detected to have mild anemia, and 23.2% (95% CI, 21.2 to 25.3) were found to have moderate to severe anemia. A cross-sectional study investigating the relationship between women’s financial decision-making power and moderate to severe anemia found that women living in households where others had decision-making control had lower odds of moderate to severe anemia than those living in households where women were the sole decision-makers [12]. This finding suggests that men and other household decisionmakers are key gatekeepers when addressing nutrition during pregnancy in rural south India.

### Pregnancy and newborn outcomes

Sixty-six women (3.9%; 95% CI, 3.9 to 5.0) reported miscarriages or stillbirths and 4.6% (95% CI, 3.6 to 5.7) infants died post-delivery. Of all newborns, 1,576 (98.0%) were born at a health facility, 123 (7.3%; 95% CI, 6.1 to 8.7) were admitted to the neonatal intensive care unit, 74 (4.5%; 95% CI, 3.5 to 5.5) had complications, and 45 (2.7%; 95% CI, 2.0 to 3.6) were hospitalized for a further work-up. Of the children of the 14 HIV-positive women, 7 (50.0%; 95% CI, 26.8 to 73.2) infants were born HIV-negative, while 3 (21.0%; 95% CI, 7.5 to 47.5) were reported as deceased in follow-up interviews. The status of the remaining 4 children (29.0%) remained unknown due to loss to follow-up. No maternal deaths were reported in the cohort.

An analysis of breastfeeding practices found that 32.3% (95% CI, 30.0 to 34.6) of women reported delayed initiation of breastfeeding. At the 6-month postpartum follow-up, 74.9% of the women reported continued breastfeeding, but only 48.5% reported exclusive breastfeeding [13]. Having more ANC visits was associated with lower odds of delayed initiation of breastfeeding and nonexclusive breastfeeding, indicating that health promotion efforts should focus on encouraging mothers to attend these visits [14]. Furthermore, this suggests that ANC visits represent an effective access point for providing breastfeeding education.

### Community engagement outcomes

The *Kisalaya* program organized 141 education and awareness events across the Mysuru *taluk*. These were attended by 5,161 community members, including 4,212 (81.6%) women and 899 (17.4%) men. Having men and key community stakeholders in attendance at these events was critical to the program’s success. More efforts are needed to encourage and include men and extended family in maternal child health programs.

### Local capacity-building outcomes

To ensure that women would continue to receive quality care even after the program ended, the *Kisalaya* program sought to build local capacity by training village health workers and health professionals in PMTCT of HIV, HIV management, and safe birthing practices. Improving local quality of care was identified as a major access point for interventions through a formative study on current birthing practices in rural India. This cross-sectional study, conducted prior to the implementation of the *Kisalaya* program’s mobile medical clinics, identified and surveyed 417 TBAs and found that only 51 (12.3%; 95% CI, 9.4 to 15.7) had heard of HIV/AIDS. Moreover, most (96.2%) had not provided their clients with ANC [15]. The study indicated that training TBAs may be beneficial for improving maternal and child health outcomes. The study also revealed the challenges of integrating TBAs into PMTCT and maternal child health programs, suggesting that training other grassroot health workers, like ASHAs and ANMs, may also be fruitful in improving health outcomes for pregnant women and infants in rural India.

In response, the *Kisalaya* program trained 40 TBAs, 72 ANMs, 127 ASHAs, and 403 CHW in preventing of vertical transmission of HIV. TBAs were also provided with standardized delivery kits in order to promote safe delivery practices in cases of emergencies when it was not possible for deliveries to take place in a hospital or healthcare setting.

## WHAT ARE THE MAIN STRENGTHS AND WEAKNESSES?

### Strengths

One of the greatest strengths of this cohort is its large sample size. As data were collected from nearly all rural villages of Mysuru *taluk*, the *Kisalaya* cohort accurately and comprehensively captured the region’s pregnancy and newborn outcomes. Furthermore, the *Kisalaya* study sample is representative of the Karnataka state population, with comparable literacy, infant mortality, maternal mortality, and HIV prevalence rates, socioeconomic status, and sex ratio between 0-6 years of age according to 2011 census data and Sample Registration System data from 2008 to 2009 [16,17]. The cohort also had a high uptake of services and excellent follow-up rates, providing a remarkably complete and rich dataset for both the prenatal and postnatal periods. In comparison to other birth cohorts in India, the *Kisalaya* cohort provides more detailed information on socio-demographic variables, making it possible to explore the impact of socio-demographic transitions over time. Additionally, the cohort offers vast amounts of information about both maternal health during the antenatal and postnatal periods and infant health outcomes, opening the door to explore the impact of maternal influences during pregnancy on children’s health outcomes. While there have not been any follow-up studies since 2012, preliminary efforts in retracing the cohort for the upcoming 2019-2020 study has been very successful, with a follow-up rate of 90% in the pilot phase of the current study.

The *Kisalaya* cohort also demonstrated the effectiveness of door-to-door recruitment methods. While 1,940 pregnancies were registered with the National Rural Health Mission, the *Kisalaya* program identified 2,211 pregnancies across the 144 villages. This means that the *Kisalaya* cohort’s methods of recruiting were successful in identifying an additional 274 (14%) pregnancies that were previously unregistered and unknown to the government of India. As a result, the *Kisalaya* program assisted in making government healthcare accessible to 274 women who otherwise would have missed opportunities to access services provided by government programs.

Another strength of the cohort is its holistic design, as the *Kisalaya* program involved community members and stakeholders, from women and their children to local health professionals. The program’s efforts to improve community awareness and build local capacity also provide an opportunity for future researchers to study the impact of health professionals and the effectiveness of these community engagement methods over time. The lessons learned from the *Kisalaya* cohort’s community engagement component were later applied to the PHRII’s 2011-2014 birth cohort, Saving Children, Improving Lives.

### Weaknesses

One of the main weaknesses of this cohort is the lack of follow-up data between 2012 and 2019. As a solution to this issue, we are currently in the process of gathering information related to the mothers and children during this period through Maternal and Child Health (MCH) cards issued universally by the government to the mothers during the antenatal period for regular follow-ups and update of information related to the mothers and children. The MCH card contains information on the child’s immunization and growth monitoring during the first 5 years of life. In the current study, we are requesting the mothers to share their MCH cards and are updating the information in the cohort database. However, it is possible that not all mothers may have their MCH cards after a decade post-delivery.

Secondly, there have been large demographic shifts along with huge transformations in terms of social, economic, and cultural aspects and health programs. Notable changes over the last decade include increased levels of rural to urban migration, changes in the social support system with the increase in nuclear families, reductions in mortality, improvements in literacy, women’s empowerment, gender equality, standard of living, and better access to healthcare provided by the government of India through various insurance and health schemes (especially in relation to MCH). However, all children who are presently in the study belonged to the *Kisalaya* cohort and would have been exposed to the same changes during their growth and development; therefore, this issue does not affect the overall credibility of the conclusions drawn through this cohort.

## DATA ACCESSIBILITY

The study data are not freely available, but the *Kisalaya* cohort team would welcome collaborations with other researchers. Data set IV (Table 1) will be made available for those participating in the ongoing study. For further information, contact Dr. Purnima Madhivanan, who is based at the Mel & Enid Zuckerman College of Public Health, University of Arizona, Tucson, AZ, USA (pmadhivanan@email.arizona.edu).

## Figures and Tables

**Table 1.** Details of the data collected and to be collected in the follow-up study of the *Kisalaya* cohort (2008-2012)

Dataset		Data collected during the *Kisalaya* study between 2008 and 2012		
Data set I		Socio-demographic data	Age, education, religion, marital status, children	
	2008-2011 (n=1,675)	Economic status	Socioeconomic class, occupation	
	Data collected from pregnant women	Sexually transmitted and blood-borne infection information	History of STI; Myths, stigma, and perceptions related to HIV/STI; Disclosure of HIV results; How HIV-positive status would change their life	
		Details of the main partner	Age, education, occupation, history of sexual risk behavior, drug and alcohol use history	
		Details of present pregnancy	Gestation in weeks; Details of immunizations during present pregnancy (tetanus toxoid injections); Clinical examination findings (general physical examination, weight, blood pressure); Perceived stress	
		Details of obstetric history	Total number of previous pregnancies; Age at first pregnancy; Outcome of each pregnancy; History of at-risk pregnancy (early primipara, multiple pregnancy, vaginal bleeding, pelvic mass, etc.); History of STI; History of abortions/stillbirth/low-birth-weight infant; History of diabetes mellitus, hypertension, cardiac diseases, Rh incompatibility	
		Laboratory investigations	Blood investigations	
				HIV, hepatitis B, syphilis, blood grouping, Rh typing, hemoglobin levels, blood sugar
			Urine examination	
				Urine albumin
			Vaginal swabs	
				Bacterial vaginosis
Data set II		Details of breastfeeding	Timing for initiation, duration, reason for not exclusively breastfeeding, etc.	
	2008-2012 (n=1,656)	Details of birth preparedness	Place of delivery, transportation, financial security in case of emergency, etc.	
	Participants were mothers in the postnatal period	Details of the child born	Living/stillbirth, age of the child if living, sex, birth weight, admission to the intensive care unit post-delivery; Any complication in the baby within 24 hr, a day to a week, or after a week (including jaundice, fever, convulsions, breathing difficulty, hypothermia, etc.)	
		Details of delivery of the baby	How many days before or after the expected date of delivery, who conducted the delivery, where was baby delivered, mode of delivery, complications, blood transfusion received, etc.	
Data set III				
	2008-2012 (n=1,638)	Details of the child	Is the baby alive, history of ill health, immunization history	
	Participants were mothers with their 6-mo-old infants	Details of breastfeeding	Is the infant still breastfeeding, duration, reason for not exclusively breastfeeding, etc.	
		Mother’s health	If currently pregnant, was it a planned pregnancy?; Birth control details; History of domestic violence, mental health including postpartum depression	
Data set IV				
	2019-2020 (n=1,544)	Anthropometry	To assess mental health	
	Data collected from adolescents between 10-12 yr and to be related with the secondary data available from the Kisalaya cohort	Height, weight, waist circumference, hip circumference	Wechsler’s Intelligence Scale for Children for assessment of cognition; Patient Health Questionnaire- adolescent for assessment of depression	
		To assess stressful life events	Adolescent life events stress scale	
		To screen vision	Snellen chart	
		To screen hearing	Audiometry	

STI, sexually transmitted infections; HIV, human immunodeficiency virus; Rh, rhesus.
